# Hepatocyte-Specific PEX16 Abrogation in Mice Leads to Hepatocyte Proliferation, Alteration of Hepatic Lipid Metabolism, and Resistance to High-Fat Diet (HFD)-Induced Hepatic Steatosis and Obesity

**DOI:** 10.3390/biomedicines12050988

**Published:** 2024-04-30

**Authors:** Xue Chen, Long Wang, Krista L. Denning, Anna Mazur, Yujuan Xu, Kesheng Wang, Logan M. Lawrence, Xiaodong Wang, Yongke Lu

**Affiliations:** 1 Department of Biomedical Sciences, Joan C. Edwards School of Medicine, Marshall University, 1700 3rd Avenue, Huntington, WV 25755, USA; chenx@marshall.edu (X.C.); mazura@marshall.edu (A.M.); 2 Department of Pathology, Guiqian International General Hospital, 1 Dongfeng Ave., Wudang, Guiyang 550018, China13124611270@163.com (Y.X.); 3 Department of Pathology, Joan C. Edwards School of Medicine, Marshall University, 1 John Marshall Drive, Huntington, WV 25755, USA; haught5@marshall.edu (K.L.D.);; 4 Department of Family and Community Health, School of Nursing, Health Sciences Center, West Virginia University, Morgantown, WV 26506, USA; kesheng.wang@hsc.wvu.edu

**Keywords:** peroxisomes, bile acid, fatty acid oxidation

## Abstract

Obesity results in hepatic fat accumulation, i.e., steatosis. In addition to fat overload, impaired fatty acid β-oxidation also promotes steatosis. Fatty acid β-oxidation takes place in the mitochondria and peroxisomes. Usually, very long-chain and branched-chain fatty acids are the first to be oxidized in peroxisomes, and the resultant short chain fatty acids are further oxidized in the mitochondria. Peroxisome biogenesis is regulated by peroxin 16 (PEX16). In liver-specific PEX16 knockout (*Pex16^Alb-Cre^*) mice, hepatocyte peroxisomes were absent, but hepatocytes proliferated, and liver mass was enlarged. These results suggest that normal liver peroxisomes restrain hepatocyte proliferation and liver sizes. After high-fat diet (HFD) feeding, body weights were increased in PEX16 floxed (*Pex16^fl/fl^*) mice and adipose-specific PEX16 knockout (*Pex16^AdipoQ-Cre^*) mice, but not in the *Pex16^Alb-Cre^* mice, suggesting that the development of obesity is regulated by liver PEX16 but not by adipose PEX16. HFD increased liver mass in the *Pex16^fl/fl^* mice but somehow reduced the already enlarged liver mass in the *Pex16^Alb-Cre^* mice. The basal levels of serum triglyceride, free fatty acids, and cholesterol were decreased, whereas serum bile acids were increased in the *Pex16^Alb-Cre^* mice, and HFD-induced steatosis was not observed in the *Pex16^Alb-Cre^* mice. These results suggest that normal liver peroxisomes contribute to the development of liver steatosis and obesity.

## 1. Introduction

Obesity is closely linked with metabolic dysfunction-associated steatotic liver disease (MASLD), previously known as non-alcoholic fatty liver disease (NAFLD), which starts from steatosis (fatty liver) [1]. When adipose triglyceride (TG) is degraded into free fatty acids (FFA) and released into the blood, the liver will reabsorb the FFA for the re-synthesis of TG, which binds to apolipoprotein B (apo B) to be released into the blood in the form of very low-density lipoprotein (VLDL). If the resynthesized TG is accumulated in the liver, then lipid droplets are formed, and steatosis is developed [2]. The early stages of steatosis are primarily asymptomatic. However, long-term fatty liver has a progression from steatosis to steatohepatitis, fibrosis, and cirrhosis, leading to hepatocellular carcinoma [1]. The impairment of fatty acid β-oxidation (FAO) is one of major reasons for the development of steatosis. Mitochondria are known as major organelles for FAO. Peroxisomes are another type of organelles for FAO [3]. Usually, very long-chain and branch chain fatty acids are oxidized in peroxisomes and the resultant shorter chain fatty acids will be further oxidized in the mitochondria. The first reaction of peroxisomal FAO is catalyzed by the rate-limiting enzyme acyl-CoA oxidase (ACOX), which produces hydrogen peroxide (H_2_O_2_) as a byproduct, and the generated H_2_O_2_ is locally decomposed by peroxisomal catalase [4].

It is well known that the peroxisome proliferator-activated receptor α (PPARα) regulates peroxisomal FAO [5,6], and the PPARα agonist WY-14,643 prevents hepatic TG accumulation in diet-induced obese mice or ethanol-fed mice [7,8]. The global or organ-specific abrogation of *pparα* deteriorated abnormal lipid metabolism in mice [9]. PPARα agonist prevented obesity in *ob*/*ob* obese mice [10], and *pparα* absence in *ob*/*ob* obese mice, making the mice more obese [11], suggesting that PPARα protects against obesity in mice. PPARα also regulates FAO and protects against hepatic steatosis in patients. For example, the expression of PPARα in human liver is reduced in NAFLD patients and negatively correlates with NAFLD severity [12]. Interestingly, in the mice lacking cytochrome P450 2A5 (CYP2A5), i.e., *cyp2a5^−/−^* mice, we observed an elevated basal level of PPARα, but more severe ethanol-induced steatosis. When the *pparα^−/−^*/*cyp2a5^−/−^* mice are created to abrogate the upregulated PPARα, ethanol-induced steatosis was further enhanced [13]. Similarly, HFD-induced steatosis was more pronounced, and evident liver necroinflammation and fibrosis were observed in the *pparα^−/−^*/*cyp2a5^−/−^* mice, but not in the *cyp2a5^−/−^* mice or the *pparα^−/−^* mice [14,15], suggesting that PPARα is still protective against the HFD-induced steatosis, steatohepatitis, and fibrosis in the *cyp2a5^−/−^* mice.

PPARα also regulates peroxisome proliferation in the liver, and consistently, PPARα agonists-induced peroxisome proliferation was not observed in the *pparα^−/−^* mice [16]. Peroxisomes can be generated de novo by budding from endoplasmic reticulum (ER). Recently, it was reported that newly born peroxisomes are a hybrid of mitochondria- and ER-derived pre-peroxisomes [17]. Peroxisomes require a group of proteins called peroxins (PEX) for their assembly and division. PEX16 is an integral membrane protein and acts as a docking site to recruit PEX3. PEX3 is a docking receptor for PEX19, and PEX19 is an import receptor for newly synthesized peroxisomal membrane proteins (PMP) [18,19]. Thus, PEX16 plays a pivotal role in the peroxisome biogenesis [20]. The loss of PEX16 results in the complete absence of any peroxisomal structures in patients [21]. In the hepatocyte-specific PEX16 knockout mice (*Pex16^Alb-Cre^*), peroxisome marker PMP70 was absent [22], indicating that peroxisomes are also absent. In this study, we utilized the *Pex16^Alb-Cre^* mice to examine the effects of liver peroxisomes on HFD-induced alterations in hepatic lipid metabolism, steatosis, and the development of obesity.

## 2. Materials and Methods

### 2.1. Studies in Experimental Rodent Models

Mice and Treatment: *Pex16*-floxed mice (*Pex16^fl/fl^* mice; purchased from Jackson Laboratory, Bar Harbour, ME, USA, strain number #034155) were crossed with transgenic mice expressing *Albumin-Cre* recombinase (Alb-Cre mice; purchased from Jackson Laboratory, Bar Harbour, ME, USA, strain number #003574) and/or expressing *adiponectin-Cre* recombinase (adipoQ-Cre mice; purchased from Jackson Laboratory, Bar Harbour, ME, USA, strain number #028020) to create the *Pex16^Alb-Cre^* mice and adipose-specific PEX16 knockout (*Pex16^AdipoQ-Cre^*) mice, respectively. The littermates *Pex16^fl/fl^* mice, not expressing Alb-Cre or AdipoQ-Cre, were used as normal control mice. All the mice were housed in temperature-controlled animal facilities with 12 h light/dark cycles and were permitted consumption of tap water and Purina standard chow ad libitum. The mice received humane care. All in vivo experiments were approved by the Institution Committee of Animal Use and Care (IACUC) at Marshall University.

The HFD and control diet (CD) were purchased from Bio-Serv company (Frenchtown, NJ, USA). HFD and the CD contained the same amount (20.5%) of protein. In HFD, 60% calories are from fat, but in CD only 16% calories are from fat.

Six to eight week-old male mice were used in this study. In Experiment 1, the *Pex16^fl/fl^* mice, *Pex16^AdipoQ-Cre^* mice, and *Pex16^Alb-Cre^* mice were fed HFD for 9 weeks to examine the effects of adipose PEX16 and hepatocyte PEX16 on HFD-induced body weight gain and steatosis. In Experiment 2, the *Pex16^fl/fl^* mice and *Pex16^Alb-Cre^* mice were fed CD and HFD for 9 weeks to further examine the effects of liver peroxisomes on HFD-induced alterations in hepatic lipid metabolism, steatosis, and the development of obesity. All mice were permitted consumption of tap water and HFD or CD ad libitum. Body weight and diet consumption were measured weekly. The amount of diet consumption was not significantly different between the HFD and CD, in either the *Pex16^fl/fl^* mice or *Pex16^Alb-Cre^* mice.

Glucose tolerance test: After 9 weeks of HFD feeding, the mice were subjected to an overnight fast (15 h), followed by glucose injection intraperitoneally at 1 g/kg. Blood was collected from their tails before glucose injection for measuring basal levels of blood glucose using a Bayer Contour blood glucose meter. After glucose injection, tail blood was collected at 30, 60, 90, and 120 min for glucose assays, respectively.

Liver pathology: After another 6 days of feeding (totaling 10 weeks), the mice were sacrificed following an overnight fast (15 h). Blood was collected for the isolating serum. The livers and gonadal white adipose tissues were collected and weighted, and the liver index and fat index were calculated as liver weight/100 g body weight and gonadal adipose tissue weight/100 g body weight. One piece of liver and gonadal adipose tissue from each mouse were put in Neutral Formalin Buffer for preparing paraffin sections for Hematoxylin and Eosin (H&E) staining and immunohistochemical staining (IHC). Liver sections with H&E staining were used for pathological evaluation. The criteria for the grade of steatosis are based on the percentage of hepatocytes containing lipid droplets: 0, none; 1, <5%; 2, 5–33%; 3, 34–66%; 4, >67%. For IHC, a Broad Spectrum IHC Select^®^ HRP/DAB kit (from EMD Millipore, Burlington, MA, USA, Cat#: DAB150) was used. Five 100× fields per liver (one 100× field area = 2.54 mm^2^) were examined for the quantification of positive staining.

Biochemical assays and Western Blotting analysis: The liver tissue aliquots were stored at −80 °C. The liver tissues were homogenized in 0.15 M potassium chloride (KCL) to make homogenates for TG contents and Western blotting analyses. The isolated serum was used for measuring TG, FFA, β-hydroxybutyrate, bile acid, glucose, and cholesterol. The commercially available assay kits and primary antibodies are listed in Table 1.

Statistical analysis: Results are expressed as mean ± S.D. Statistical evaluation was carried out using two-way analysis of variance (ANOVA) with subsequent use of the Student-Newman-Keuls post hoc test. For body weight changes, Repeated Measures ANOVA was carried out. *p* < 0.05 was considered as statistical significance.

### 2.2. Studies in Patients with Chronic Liver Diseases

Liver paraffin sections from patients with primary chronic liver diseases, including gallstone, cholecystitis, hepatic cyst, hepatic hemangioma, cirrhosis, and hepatocellular carcinoma, were collected from the Guiqian International General Hospital, Guiyang, China in the past three years. The adjacent or distal tissues of the original liver lesions were blindly diagnosed and scored by a pathologist (L.W.). In total, 39 cases with a typical spectrum of MASLD (steatosis, inflammation, fibrosis) were selected for IHC. Primary antibodies were purchased from international suppliers: ACOX-1 (1:300, Atlas Antibodies, Stockholm, Sweden); PEX16, PMP70, and Catalase (1:500, Bioss Antibodies, Beijing, China); Cyclooxygenase 2 (1:500, Abcam, Boston, MA, USA); MDA (1:100, Kanglang Biotechology, Shanghai, China). IHC UltraView Univeral DAB kit was used to detect above markers by polymer method at Ventana ULTRA automatic instrument (Roche Diagnostics, Rotkreuz, Switzerland). Heat-mediated antigen retrieval was performed with epitope retrieval solution Tris-EDTA buffer (pH 9.0), and hematoxylin staining was used as a counterstain.

## 3. Results

### 3.1. The Resistance to HFD-Induced Body Weight Gain Is Observed in the Pex16^Alb-Cre^ Mice but Not in the Pex16^AdipoQ-Cre^ Mice

**Figure 1:**
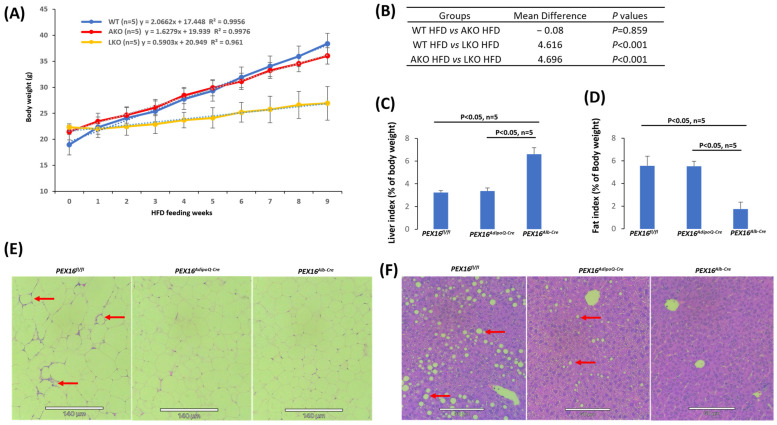
HFD-induced body weight gain in the *Pex16^fl/fl^* mice and *Pex16^AdipoQ-Cre^* mice but not in the *Pex16^Alb-Cre^* mice. (**A**) Body weight gain in the HFD-fed mice (n = 5); (**B**) Repeated Measures ANOVA analysis; (**C**) Liver index (n = 5); (**D**) Fat index (n = 5); (**E**) H&E staining showing adipose inflammation as indicated by “Crown”. The images are representatives of the 5 mice. (**F**) H&E staining showing lipid droplets (Arrows) in liver sections. The images are representatives of the 5 mice. WT, *Pex16^fl/fl^* mice; AKO, adipose-specific PEX16 knockout (*Pex16^AdipoQ-Cre^*) mice; LKO, liver-specific PEX16 knockout (*Pex16^Alb-Cre^*) mice.

The male *Pex16^fl/fl^* mice, *Pex16^AdipoQ-Cre^* mice (AKO), and *Pex16^Alb-Cre^* mice (LKO mice) were fed HFD, and their body weights were measured on a weekly basis. As shown in Figure 1A, the HFD-induced body weight gain gradually increased with a linear relationship in all groups. The slope was about 2.0, 1.6, 0.6 for the *Pex16^fl/fl^* mice, *Pex16^AdipoQ-Cre^* mice, and *Pex16^Alb-Cre^* mice, respectively. We used Repeated Measures ANOVA to compare the 3 groups (Figure 1B). Body weights were increased in the *Pex16^Alb-Cre^* mice to a much lesser extent than in the *Pex16^fl/fl^* mice and the *Pex16^AdipoQ-Cre^* mice, but there was no difference in the HFD-induced body weight gain between the *Pex16^fl/fl^* mice and the *Pex16^AdipoQ-Cre^* mice, suggesting that liver PEX16, but not adipose PEX16, has a significant influence on HFD-induced body weight gain. Liver mass, as indicated by the liver index, was comparable in the *Pex16^fl/fl^* mice and the *Pex16^AdipoQ-Cre^* mice, but it was much higher in the *Pex16^Alb-Cre^* mice (Figure 1C). The gonadal adipose tissue mass, as indicated by fat index, was also comparable in the *Pex16^fl/fl^* mice and the *Pex16^AdipoQ-Cre^* mice, but it was much lower in the *Pex16^Alb-Cre^* mice (Figure 1D). The H&E staining in the sections of adipose tissues showed that the adipocytes in the HFD-fed *Pex16^fl/fl^* mice were about 50–60 µm in diameter, which was larger than those in the HFD-fed *Pex16^AdipoQ-Cre^* mice and *Pex16^Alb-Cre^* mice (about 30–40 µm in diameter), and the inflammation as indicated by the “Crown” formation was observed in the HFD-fed *Pex16^fl/fl^* mice, but not in the HFD-fed *Pex16^AdipoQ-Cre^* mice and *Pex16^Alb-Cre^* mice (Figure 1E). Interestingly, HFD-induced steatosis, as indicated by lipid droplet formation, was observed in the *Pex16^AdipoQ-Cre^* mice to a lesser extent than in the *Pex16^fl/fl^* mice, but steatosis was almost not detectable in the *Pex16^Alb-Cre^* mice (Figure 1F). Steatosis grades, based on the percentage of hepatocytes containing lipid droplets (scale 0–4), for the *Pex16^fl/fl^* mice, *Pex16^AdipoQ-Cre^* mice, and *Pex16^Alb-Cre^* mice were 2.9 ± 0.61, 1.2 ± 0.36, and 0.25 ± 0.67, respectively. These results suggest that the absence of adipose PEX16 does not have an influence on HFD-induced obesity, although HFD-induced steatosis is partially attenuated; in contrast, the absence of liver PEX16 leads to a resistance to HFD-induced body weight gain and HFD-induced steatosis.

The interesting observation in the *Pex16^Alb-Cre^* mice that the liver index was increased and the fat index was decreased inspired us to further investigate the potential interaction between liver and adipose tissues by evaluating the HFD-induced obesity and hepatic lipid metabolism in the *Pex16^fl/fl^* mice and *Pex16^Alb-Cre^* mice.

### 3.2. HFD Feeding Induces Obesity in the Pex16^fl/fl^ Mice but Not in the Pex16^Alb-Cre^ Mice

**Figure 2:**
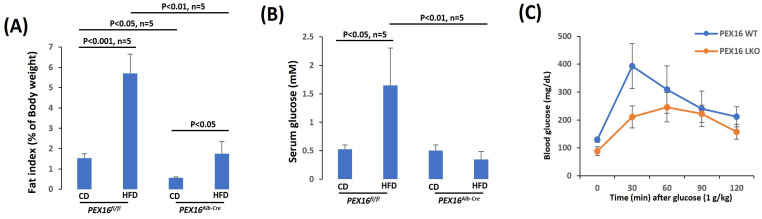
HFD-induced glucose intolerance in the *Pex16^fl/fl^* mice but not in the *Pex16^Alb-Cre^* mice. (**A**) HFD-induced gonadal adipose tissue expansion as indicated by fat index (n = 5); (**B**) HFD-induced hyperglycemia (n = 5); (**C**) Glucose tolerance test (n = 3).

Interestingly, the basal levels of gonadal adipose tissue mass (fat index) were lower in the *Pex16^Alb-Cre^* mice than in the *Pex16^fl/fl^* mice, and in response to the HFD feeding the gonadal adipose tissues were expanded to a greater extent in the *Pex16^fl/fl^* mice than in the *Pex16^Alb-Cre^* mice (Figure 2A). Like the HFD-induced body weight gain, HFD-induced hyperglycemia in the *Pex16^fl/fl^* mice but not in the *Pex16^Alb-Cre^* mice (Figure 2B). Glucose tolerance test showed an intolerance to the bolus injection of glucose in the *Pex16^fl/fl^* mice to a greater extent than in the *Pex16^Alb-Cre^* mice (Figure 2C). These results suggest that HFD induces glucose intolerance in the *Pex16^fl/fl^* mice rather than in the *Pex16^Alb-Cre^* mice.

### 3.3. The Absence of Liver PEX16 Leads to Hepatocyte Proliferation

**Figure 3:**
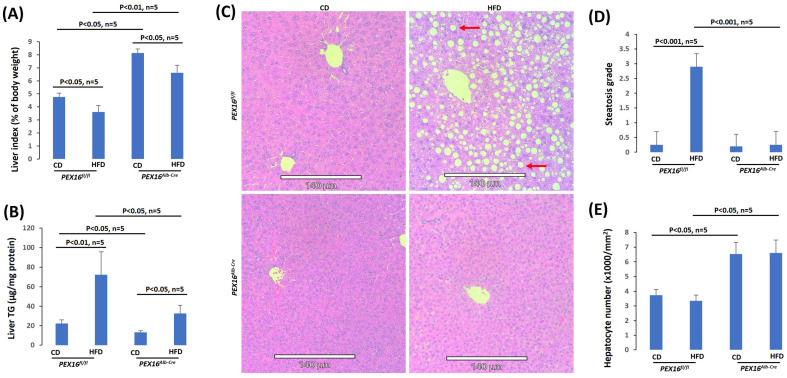
HFD-induced steatosis in the *Pex16^fl/fl^* mice but not in the *Pex16^Alb-Cre^* mice. (**A**) Liver index (n = 5); (**B**) Liver TG contents (n = 5); (**C**) H&E staining showing lipid droplets (Arrows) in liver sections; (**D**) Steatosis quantification (n = 5); (**E**) Hepatocyte nuclear number (n = 5).

In contrast to gonadal adipose tissues, basal levels of liver mass (liver index) were lower in the *Pex16^fl/fl^* mice than in the *Pex16^Alb-Cre^* mice, and HFD feeding lowered the liver index in both the *Pex16^fl/fl^* mice and *Pex16^Alb-Cre^* mice although it was still higher in the *Pex16^Alb-Cre^* mice than in the *Pex16^fl/fl^* mice (Figure 3A). The mechanisms by which HFD lowered the liver index are different in the *Pex16^Alb-Cre^* mice than in the *Pex16^fl/fl^* mice. In the *Pex16^fl/fl^* mice, both the body weight and liver weight were increased by the HFD feeding, but the body weights increased (Week 0:18.96 ± 2.21 g vs. Week 10: 39.44 ± 2.78 g) to a greater extent than liver weights did (CD: 0.99 ± 0.08 g vs. HFD: 1.7 ± 0.26 g), so liver index was lowered. However, in the *Pex16^Alb-Cre^* mice, after the HFD feeding, liver weights were not increased but somehow decreased (CD: 2.37 ± 0.013 g vs. HFD: 1.75 ± 0.22 g), and body weights were almost not changed (Week 0: 22.48 ± 0.46 g vs. Week10: 27.62 ± 3.62 g). Interestingly, liver TG contents were increased by the HFD feeding in the *Pex16^fl/fl^* mice to a greater extent than in the *Pex16^Alb-Cre^* mice (Figure 3B). In the H&E staining liver sections, while HFD-induced lipid droplet formation were observed in the *Pex16^fl/fl^* mice but not in the *Pex16^Alb-Cre^* mice (Figure 3C,D), very interestingly, the sizes of hepatocyte cell nucleus in the *Pex16^Alb-Cre^* mice were smaller than in the *Pex16^fl/fl^* mice, and correspondingly, the number of hepatocyte cell nucleus per mm^2^ was higher in the *Pex16^Alb-Cre^* mice than in the *Pex16^fl/fl^* mice (Figure 3E).

**Figure 4:**
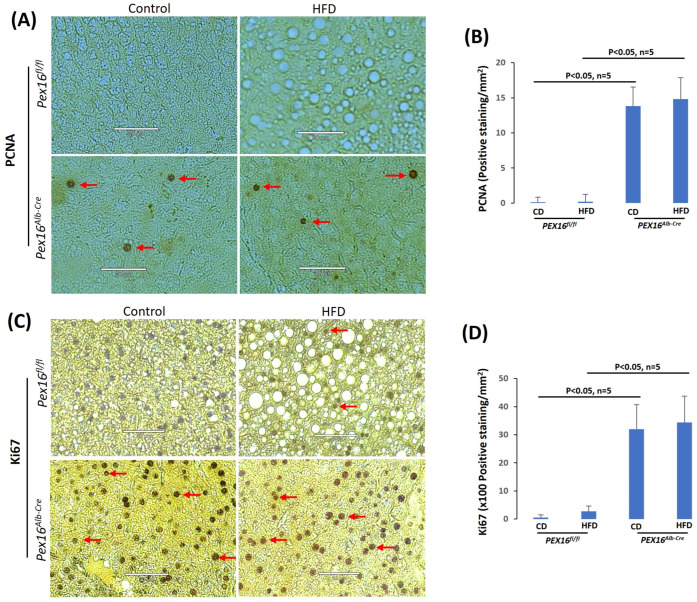
The absence of liver PEX16 leads to hepatocyte proliferation. (**A**) PCNA positive staining was observed in the *Pex16^Alb-Cre^* mice, but not in the *Pex16^fl/fl^* mice. Arrows show representative positive staining. The images are representative of the 5 mice. (**B**) PCNA staining quantification (n = 5). (**C**) Ki67 staining was observed in the *Pex16^Alb-Cre^* mice, but not in the *Pex16^fl/fl^* mice. Arrows show representative positive staining. The images are representative of the 5 mice. (**D**) Ki67 staining quantification (n = 5). Arrows show representative positive staining.

To check whether the increase in hepatocyte nuclear number results from hepatocyte proliferation, cell proliferation markers proliferating cell nuclear antigen (PCNA) and Ki67 were detected by IHC. PCNA staining was negative and HFD did not induce PCNA in the *Pex16^fl/fl^* mice, but it was positive in the *Pex16^Alb-Cre^* mice fed with CD or HFD (Figure 4A,B). Ki67 staining was negative in the CD-fed *Pex16^fl/fl^* mice, but HFD slightly induced Ki67 positive staining in the *Pex16^fl/fl^* mice. Ki67 was positively stained in the *Pex16^Alb-Cre^* mice, but HFD did not further increase the Ki67 staining (Figure 4C,D). These results suggest that the absence of PEX16 leads to hepatomegaly and hepatocyte proliferation, but HFD has no effect on the hepatocyte proliferation.

### 3.4. The Absence of Liver PEX16 Leads to the Alteration of Fatty Acid Metabolism in the Liver

**Figure 5:**
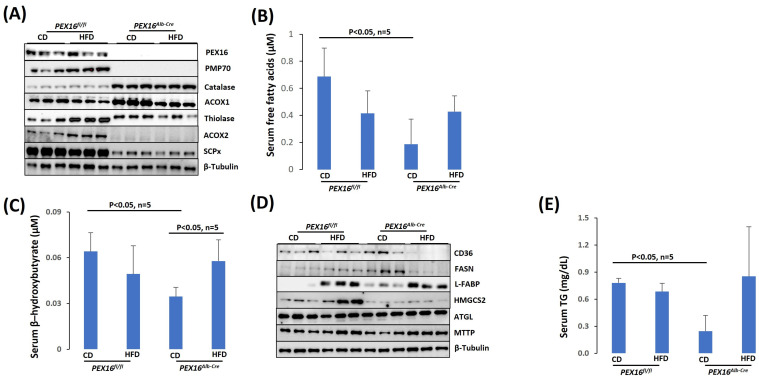
The absence of liver PEX16 lowered serum-free fatty acids, ketone bodies and TG. (**A**) Expression of liver peroxisomal fatty acid β-oxidation enzymes; (**B**) Serum fatty acids (n = 5); (**C**) Serum β-hydroxybutyrate (n = 5); (**D**) Expression of fat metabolism enzymes; (**E**) Serum TG (n = 5).

In the *Pex16^Alb-Cre^* mice, the absence of PEX16 was confirmed by Western blotting analysis (Figure 5A). Peroxisome marker PMP70 was undetectable, indicating the absence of peroxisomes in the *Pex16^Alb-Cre^* mice. In the peroxisomal matrix, fatty acid β-oxidation enzyme ACOX1 and thiolase oxidize long straight chain fatty acids, and ACOX2 and its downstream enzyme sterol carrier protein x (SCPx) oxidize branched-chain fatty acids [23]. In the *Pex16^Alb-Cre^* mice, ACOX2 and SCPx were downregulated. However, ACOX1 and thiolase were upregulated. Aligning with the ACOX1, catalase was also upregulated in the *Pex16^Alb-Cre^* mice (Figure 5A). Consistent with the upregulation in ACOX1, thiolase and catalase, basal levels of serum fatty acids were lower in the *Pex16^Alb-Cre^* mice than in the *Pex16^fl/fl^* mice, and HFD tended to elevate serum fatty acids in the *Pex16^Alb-Cre^* mice (Figure 5B). Likewise, basal levels of serum β–hydroxybutyrate, a major component of ketone bodies, were also lower in the *Pex16^Alb-Cre^* mice, and HFD tended to elevate serum β–hydroxybutyrate (Figure 5C). FFA is absorbed through fatty acid transporters CD36 or synthesized by fatty acid synthase (FASN), and transferred by liver fatty acid-binding protein (L-FABP) [24]. Basal CD36 expression was not changed, but the basal expressions of L-FABP and FASN were upregulated in the *Pex16^Alb-Cre^* mice (Figure 5D). In the *Pex16^Alb-Cre^* mice, HFD feeding induced L-FABP but inhibited liver CD36 and FASN. The 3-Hydroxy-3-methylglutaryl-CoA synthase-2 (HMGCS2) mediates the rate-limiting step in mitochondrial synthesis of ketone bodies [25], but HMGCS2 was downregulated in the *Pex16^Alb-Cre^* mice (Figure 5D). ATGL (adipose triglyceride lipase) is responsible for the initial step of TG degradation [26], and MTTP (microsomal triglyceride transfer protein) transfers newly synthesized TG in the ER for the assembly of VLDL [27]. The basal levels of ATGL and MTTP were not changed (Figure 5D), although basal serum TG levels were lower in the *Pex16^Alb-Cre^* mice than in the *Pex16^fl/fl^* mice (Figure 5E).

### 3.5. The Absence of Liver PEX16 Leads to the Alteration of Cholesterol and Bile Acid Metabolism

**Figure 6:**
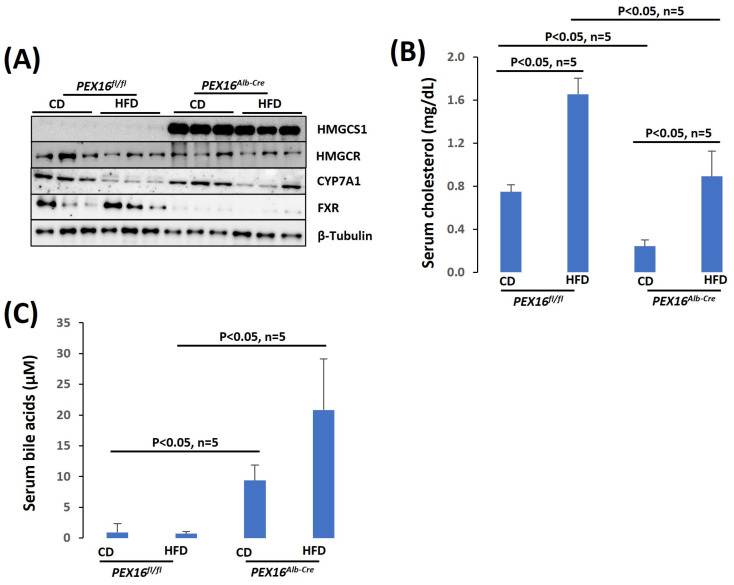
The absence of liver PEX16 altered cholesterol and bile acid metabolism. (**A**) Expression of liver cholesterol and bile acid synthetic enzymes; (**B**) Serum cholesterol (n = 5); (**C**) Serum bile acids (n = 5).

HMGCS1 is upregulated in the *Pex16^Alb-Cre^* mice (Figure 6A). Unlike HMGCS2, which is located in the mitochondria for the synthesis of ketone bodies, HMGCS1 is used for cholesterol synthesis [28]. The 3-Hydroxy-3-methylglutaryl-CoA reductase (HMGCR) is a rate limiting enzyme of cholesterol synthesis [29]. HMGCR was not altered in the *Pex16^Alb-Cre^* mice, but the basal levels of serum cholesterol were lower in the *Pex16^Alb-Cre^* mice than in the *Pex16^fl/fl^* mice (Figure 6B). Cholesterol is used for bile acid synthesis [30]. Correspondingly, the serum levels of bile acid were higher in the *Pex16^Alb-Cre^* mice than in the *Pex16^fl/fl^* mice (Figure 6C). CYP7A1 is a rate limiting enzyme that catalyzes bile acid synthesis [30], but CYP7A1 was not changed in the *Pex16^Alb-Cre^* mice [30]. As an intracellular bile acid sensor, the nuclear receptor farnesoid X receptor (FXR) is critical for bile acid and lipid homeostasis [31]. FXR was almost undetectable in the *Pex16^Alb-Cre^* mice (Figure 6C), implying that the elevation of serum bile acids is associated with the impaired regulation in FXR on bile acid homeostasis.

### 3.6. Peroxisomes are Associated with Liver Steatosis in Patients with Typical Spectrum of MASLD

**Figure 7:**
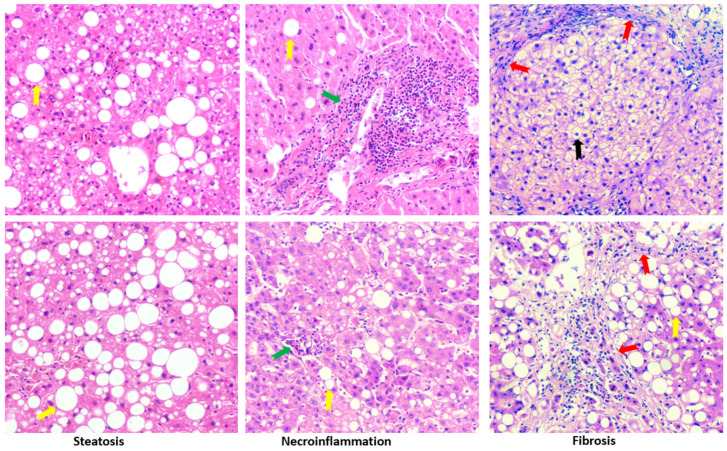
H&E staining in the liver sections from patients with chronic liver diseases. Yellow arrows, green arrows, and red arrows indicate lipid droplets, inflammation foci, and fibrosis, respectively. Black arrows show ballooning degeneration. The images are representatives of the 37 patients with chronic liver diseases.

**Figure 8:**
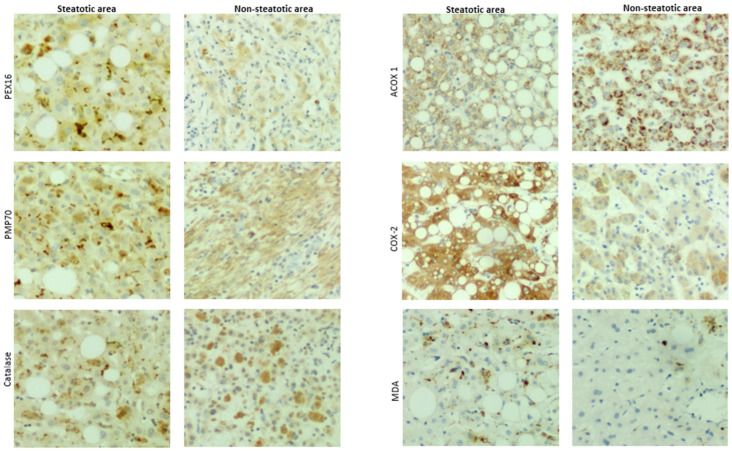
IHC staining in liver sections from patients with chronic liver diseases. The images are representatives of the 37 patients with chronic liver diseases.

In addition to cirrhosis and hepatocellular carcinoma, which are classified in MASLD, primary chronic liver diseases like gallstone, cholecystitis, hepatic cyst, hepatic hemangioma also exhibited typical spectrum of MASLD, i.e., steatosis, steatohepatitis and fibrosis. In adjacent tissues and distal tissues of the original lesions, ballooning degeneration, steatosis, necroinflammation, and fibrosis were observed (Figure 7). The patients’ liver sections displaying typical spectrum of MASLD were selected to study the association of peroxisomes with liver steatosis. IHC for peroxisomal membrane proteins (PEX16 and PMP70) and peroxisomal matrix enzymes (catalase and ACOX1) were performed in these liver sections. As shown in Figure 8, PEX16 and PMP70 were stained much stronger in the area full of lipid droplets (steatotic area) than in the area without lipid droplets (non-steatotic area), while catalase was identically stained regardless of lipid droplets. Interestingly, ACOX1 staining was weaker in the steatotic area than in the non-steatotic area, suggesting that steatosis is associated with the impaired peroxisomal fatty acid β-oxidation.

Here, we also detected inducible cyclooxygenase 2 (COX-2) because COX-2-produced prostaglandins is a class of substrates of ACOX1, and COX-2/ACOX1 formed an alternate pathway for fatty acid β-oxidation [32]. Interestingly, in contrast to ACOX1, COX-2 staining was much stronger in the steatotic area than in the non-steatotic area. Thus, under the ACOX1 suppression, the induced COX-2 may increase the accumulation of prostaglandins and promote inflammation and lipid peroxidation [33]. Indeed, necroinflammation, along with lipid droplets, was observed in Figure 7, and lipid peroxidation marker malondialdehyde (MDA) was mainly detected in the steatotic area (Figure 8). These results suggest that the suppressed ACOX1, but not the increased peroxisomal membrane proteins PEX16 and PMP70, leads to steatosis and the subsequent advancement to necroinflammation in the liver.

## 4. Discussion

There is an interaction between the liver and adipose tissues. It is well known that obesity leads to hepatic steatosis. In this study, we investigated the effects of liver peroxisomal function on the development of diet-induced obesity. We found that HFD-induced body weight gain, adipose tissue expansion, and glucose intolerance were observed in the *Pex16^fl/fl^* mice rather than in the *Pex16^Alb-Cre^* mice that lack structural peroxisomes, and consistently, HFD-induced steatosis was observed in the *Pex16^fl/fl^* mice but not in the *Pex16^Alb-Cre^* mice. Interestingly, the *Pex16^Alb-Cre^* mice displayed lower basal levels of serum TG, cholesterol, free fatty acids, and ketone body, but serum levels of bile acids were higher in the *Pex16^Alb-Cre^* mice. Additionally, the *Pex16^Alb-Cre^* mice exhibited elevated hepatocyte proliferation, which needs more cholesterol and energy (ATP) to construct cell membrane. These results suggest that the absence of liver PEX16 and peroxisomes inhibits the development of obesity through enhancing hepatocyte proliferation and increasing hepatic lipid consumption.

When we observed a difference in HFD-induced body weight gain between the *Pex16^Alb-Cre^* mice and the *Pex16^fl/fl^* mice, we did not observe a difference between the *Pex16^AdipoQ-Cre^* mice and the *Pex16^fl/fl^* mice, implying that it is liver peroxisomes, but not adipose peroxisomes, that affect HFD-induced obesity. Another interesting observation is that HFD-induced steatosis was minor in the *Pex16^AdipoQ-Cre^* mice compared to the *Pex16^fl/fl^* mice. HFD-induced body weight gain and adipose tissue expansion were comparable in the *Pex16^AdipoQ-Cre^* mice and *Pex16^fl/fl^* mice, however, adipose inflammation was observed in the *Pex16^fl/fl^* mice, but not in the *Pex16^AdipoQ-Cre^* mice. It will be interesting to further address the possible relationship between adipose inflammation and the development of steatosis.

Recently, it was reported that HFD-induced obesity was developed in the *Pex16^AdipoQ-Cre^* mice to a greater extent than in the WT mice. Actually, HFD-induced body weight gain in the *Pex16^AdipoQ-Cre^* mice was not higher than in the WT mice until after 27 weeks of feeding [34]. Previously, we found in human populations that single nucleotide polymorphisms (SNP) of CYP2A6 (CYP2A5 in mice) were detected in class I obesity patients with a body mass index (BMI) of 30–34.9 but not in class II and III obesity patients with BMI > 35, implicating that CYP2A6 is associated with the early stages of obesity; correspondingly, differences in HFD-induced body weight gain between the *cyp2a5^−/−^* mice and WT mice was observed as early as 6 weeks [14]. CYP2A5/6 is also mainly expressed in the liver. Thus, it seems that the liver affects the early stage of obesity, and the expanded adipose tissues will further deteriorate obesity in later stages. This claim is supported by a recent study showing that hepatic lipid metabolism-associated genes were increased in response to HFD as early as 3 h, and liver TG contents were increased at 6 h, whereas adipose TG contents were increased after 12 h [35]. As for peroxisomes, it is possible that the absence of liver peroxisomes initiates to speed up the development of obesity, and the expanded adipose tissues further worsens the occurring obesity. Dietary fat digestion and absorption is different from proteins and carbohydrates. Proteins and carbohydrates are digested into amino acids and glucose, respectively, and then the absorbed amino acids and glucose directly enter the liver via portal vein. However, food fat (triglyceride) is digested to fatty acids and glycerol or monoglyceride in the gut lumen and then re-synthesized to triglyceride in the intestinal epithelial cells and directly absorbed into lymph instead of the blood. How the liver responds to HFD earlier than adipose tissues needs further studies.

**Figure 9:**
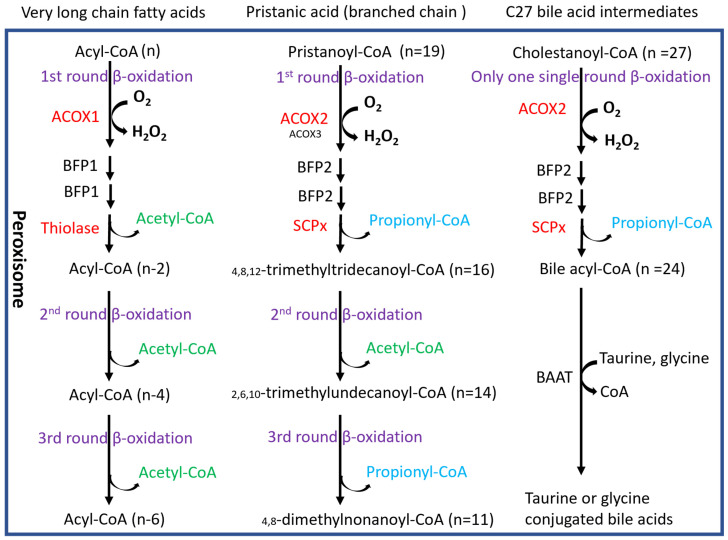
Pathways of peroxisomal β-oxidation. The small font size of ACOX3 means that ACOX3 plays a minor role in the β-oxidation compared with ACOX2. BFP, bifunctional protein; BAAT, bile acyl-CoA: amino acid acyltransferase. Enzymes in red color were measured in this study. Acetyl-CoA and Propionyl-CoA are distinguished in green and blue fonts, respectively. The rounds of β-oxidation are indicated in purple fonts.

In peroxisomes, at least two fatty acid oxidation pathways are identified (Figure 9). ACOX1 and its downstream thiolase oxidize very long-chain fatty acids, and ACOX2 and its downstream SCPx oxidize-branched-chain fatty acids [23]. Interestingly, ACOX1 is inducible by PPARα agonists, but ACOX2 is not inducible [23]. An interesting observation in the *Pex16^Alb-Cre^* mice is that ACOX1 and thiolase were upregulated but ACOX2 and SCPx were downregulated. Basal levels of serum fatty acids were lower in the *Pex16^Alb-Cre^* mice than in the *Pex16^fl/fl^* mice, which is consistent with the upregulated ACOX1 pathway. Among polyunsaturated fatty acids (PUFA), arachidonic acid is special. Very minor free arachidonic acid is detected in cells. Usually, arachidonic acid is released from phospholipid by phospholipase A2 (PLA2) and is used by COX-2 for the synthesis of prostaglandins and thromboxane, both of which are substrates of ACOX1 [36]. When the expression of COX-2 is elevated but ACOX1 is suppressed, these inflammatory mediators are supposed to be accumulated and subjected to lipid peroxidation, as we observed in human samples (Figure 8).

The downregulation in the ACOX2 pathway may lead to an accumulation of branched-chain fatty acids. Good examples of branched fatty acids are phytanic acid and pristanic acid. Pristanic acid is a 2-methyl-branched-chain fatty acid that can be directly β-oxidized by ACOX2. Phytanic acid is a 3-methyl-branched-chain fatty acid that needs to be α-oxidized to pristanic acid. Phytanic acid is derived from phytol, and phytol is derived from the plant chlorophyll. Humans obtain phytanic acid primarily from dairy products and from the fats of ruminant animals. Bacteria in the rumen of these animals can digest plant chlorophyll to release phytol, which is absorbed and further metabolized to phytanic acid by the ruminant animals. Interestingly, phytanic and pristanic acid are PPARα agonists [37]. Phytanic acid accumulation was observed in patients lacking functional peroxisomes [38,39]. Serum-free fatty acids were decreased in the *Pex16^Alb-Cre^* mice, but it cannot be ruled out that phytanic acid is accumulated in the *Pex16^Alb-Cre^* mice. Unfortunately, we could not measure the serum levels of phytanic acid. However, it is plausible that the upregulation in PPARα-regulated ACOX1 in the *Pex16^Alb-Cre^* mice is associated with phytanic acid accumulation resulted from the downregulation in ACOX2.

It is well known that bile acids are derived from cholesterol, and cholesterol is synthesized from acetyl-CoA. Acetyl-CoA produced by peroxisomal fatty acid β-oxidation can be used for cholesterol synthesis [29]. As shown in Figure 9, in addition to branched fatty acids, C27 bile acid intermediates are also oxidized by ACOX2 and ultimately matured into C24 conjugated bile acids in peroxisomes [30,40]. Interestingly, even though ACOX2 was downregulated, serum bile acids were still elevated in the *Pex16^Alb-Cre^* mice. Consistently, serum cholesterol was decreased in the *Pex16^Alb-Cre^* mice. Bile acids are metabolic signals to promote liver regeneration [41]. We observed an increased number of hepatocytes and positive staining of cell proliferation markers PCNA and Ki67 in the *Pex16^Alb-Cre^* mice, suggesting that hepatocyte proliferation occurs in the *Pex16^Alb-Cre^* mice. Thus, cholesterol-derived bile acids may promote liver regeneration, and cholesterol itself is used for cell membrane structure material for proliferated hepatocytes. Liver regeneration is critical for survival in drug-induced acute liver failure [42]. It is possible that hepatocyte proliferation is a reason why the *Pex16^Alb-Cre^* mice were resistant against the HFD-induced steatosis and obesity. As a principle of evidence, we observed thioacetamide-induced liver injury in the *Pex16^fl/fl^* mice but not in the *Pex16^Alb-Cre^* mice: serum ALT elevation, induced by thioacetamide (200 mg/kg, i.p., 18 h), was lower in the *Pex16^Alb-Cre^* mice (84.31 ± 10.18 U/L) than in the *Pex16^fl/fl^* mice (253.46 ± 44.14 U/L). Furthermore, we recently showed that the *Pex16^Alb-Cre^* mice were resistant to alcohol-induced steatosis [22].

To sum up, we show that hepatocyte-specific PEX16-absent mice exhibit hepatocyte proliferation, increased liver mass but decreased adipose tissue mass, alteration of fatty acids-cholesterol-bile acid metabolism, and resistance to HFD-induced hepatic steatosis and obesity. However, this study is only a descriptive observation, the exact mechanisms and relationship of peroxisomes with fatty acids-cholesterol-bile acid metabolism, hepatocyte proliferation, and resistance against steatosis and obesity need further studies.

## Figures and Tables

**Table 1:** Antibody assay kits and other material sources.

Name	Cat#	Company
CYP4A11 antibody	11688-1-AP	Proteintech, Rosemont, IL, USA
Catalase antibody	21260-1-AP	Proteintech, Rosemont, IL, USA
ACOX1 antibody	10957-1-AP	Proteintech, Rosemont, IL, USA
PEX16 antibody	14816-1-AP	Proteintech, Rosemont, IL, USA
ATGL antibody	55190-1-AP	Proteintech, Rosemont, IL, USA
β-Tubulin antibody	66240-1-Ig	Proteintech, Rosemont, IL, USA
CD36 antibody	18836-1-AP	Proteintech, Rosemont, IL, USA
FXR antibody	25055-1-AP	Proteintech, Rosemont, IL, USA
SCPx antibody	14397-1-AP	Proteintech, Rosemont, IL, USA
L-FABP antibody	13626-1-AP	Proteintech, Rosemont, IL, USA
FASN antibody	10624-2-AP	Proteintech, Rosemont, IL, USA
Thiolase antibody	HPA006764-25 μL	Sigma-Alderich, St. Louis, MO, USA
HMGCR antibody	SAB4200529-200 μL	Sigma-Alderich, St. Louis, MO, USA
PCNA antibody	Sc-25280	Santa Cruz Biotechnology, Dallas, TX, USA
Ki67 antibody	sc-23900	Santa Cruz Biotechnology, Dallas, TX, USA
CYP7A1 antibody	Sc-518007	Santa Cruz Biotechnology, Dallas, TX, USA
MTTP antibody	Sc-135994	Santa Cruz Biotechnology, Dallas, TX, USA
ACOX2 antibody	Sc-514320	Santa Cruz Biotechnology, Dallas, TX, USA
HMGCS1 antibody	422015	Cell Signaling, Beverly, MA, USA
HMGCS2 antibody	209405	Cell Signaling, Beverly, MA, USA
PMP70 antibody (ab3421)	ab3421	Abcam, Waltham, MA, USA
Bile acid assay	E-BC-K181-M	Elabscience, Houston, TX, USA
Cholesterol assay kit	TR13421	Thermo Fisher, Waltham, MA, USA
Triglyceride assay kit	TR22421	Thermo Fisher, Waltham, MA, USA
EnzyChrom Free fatty acid assay kit	EFFA-100	BioAssay Systems, Hayward, CA, USA
β-Hydroxybutyrate (Ketone Body) Colorimetric Assay Kit	700190	Cayman Chemical Company, Ann Harbor, MI, USA
High-fat diet	F3282	Bio-Serv, Flemington, NJ, USA
Control diet	F4031	Bio-Serv, Flemington, NJ, USA

## Data Availability

Data are contained within the article.
